# hASH1 nuclear localization persists in neuroendocrine transdifferentiated prostate cancer cells, even upon reintroduction of androgen

**DOI:** 10.1038/s41598-019-55665-y

**Published:** 2019-12-13

**Authors:** Jennifer A. Fraser, Joseph E. Sutton, Saba Tazayoni, Isla Bruce, Amy V. Poole

**Affiliations:** School of Applied Sciences, Edinburgh Napier University, Sighthill Campus, Edinburgh, EH11 4BN UK

**Keywords:** Cancer models, Mechanisms of disease

## Abstract

Neuroendocrine prostate cancer (NEPC) is thought to arise as prostate adenocarcinoma cells transdifferentiate into neuroendocrine (NE) cells to escape potent anti-androgen therapies however, the exact molecular events accompanying NE transdifferentiation and their plasticity remain poorly defined. Cell fate regulator ASCL1/hASH1’s expression was markedly induced in androgen deprived (AD) LNCaP cells and prominent nuclear localisation accompanied acquisition of the NE-like morphology and expression of NE markers (NSE). By contrast, androgen-insensitive PC3 and DU145 cells displayed clear nuclear hASH1 localisation under control conditions that was unchanged by AD, suggesting AR signalling negatively regulated hASH1 expression and localisation. Synthetic androgen (R1881) prevented NE transdifferentiation of AD LNCaP cells and markedly suppressed expression of key regulators of lineage commitment and neurogenesis (REST and ASCL1/hASH1). Post-AD, NE LNCaP cells rapidly lost NE-like morphology following R1881 treatment, yet ASCL1/hASH1 expression was resistant to R1881 treatment and hASH1 nuclear localisation remained evident in apparently dedifferentiated LNCaP cells. Consequently, NE cells may not fully revert to an epithelial state and retain key NE-like features, suggesting a “hybrid” phenotype. This could fuel greater NE transdifferentiation, therapeutic resistance and NEPC evolution upon subsequent androgen deprivation. Such knowledge could facilitate CRPC tumour stratification and identify targets for more effective NEPC management.

## Introduction

Prostate cancer is dependent on androgen for growth, therefore androgen deprivation therapy (ADT) is central to restricting prostate cancer progression and improving patient survival^1,2^. Unfortunately, resistance to ADT invariably arises and the tumour relapses as aggressive, castration-resistant prostate cancer (CRPC)^3^; a significant clinical challenge due to a lack of therapeutic targets to treat CRPC. Neuroendocrine prostate cancer (NEPC) represents an advanced, aggressive and lethal form of CRPC^4^, characterised by increased prevalence of neuroendocrine (NE)-like cells^3^. NE cells are highly resistant to therapy and secrete a variety of growth factors and hormones to fuel highly aggressive castrate resistant tumour growth^5^.

NEPC is thought to arise from transcriptional reprogramming and transdifferentiation of prostate adenocarcinoma cells into NE-like cells, rather than expansion of the small population of pre-existing prostate NE cells^3^. Transdifferentiation represents an alternative route of acquired drug resistance known as lineage plasticity, where cells switch to a lineage no longer reliant on the drug target for survival, to escape the therapeutic pressure, in this case, potent androgen inhibition^3^. Despite the severity of NEPC, there are fundamental gaps in our mechanistic understanding of NE transdifferentiation and NEPC evolution, meaning specific drug targets, and therapeutics to treat NEPC do not exist.

Prolonged androgen deprivation can seriously impact on patient quality of life by causing serious side effects, including increased risk of cardiovascular disease and stroke^6^. At risk patients receive ADT intermittently (iADT), via treatment “breaks”, to maintain androgen concentrations and limit the potentially life-limiting side-effects^7^. Transdifferentiated LNCaP prostate cancer cells can lose NE-like features upon return to control, androgen rich conditions *in vitro*, indicating NE transdifferentiation is not fixed^8,9^. However the molecular events involved in this “dedifferentiation” and whether NE-like cells within prostate tumours revert to adenocarcinoma cells during breaks in iADT, is unknown.

Altered expression of key regulators of cell fate and lineage commitment have been associated with NE transdifferentiation in prostate cancer, including ASCL1 and REST. ASCL1 (Achaete-Scute Complex-Like 1) co-ordinates extensive transcriptional reprogramming to drive neural progenitor cell differentiation and commitment to neuronal lineages^10^, whilst the master repressor of neurogenesis, REST (RE1-silencing transcription factor), prevents neurogenesis by globally repressing expression of hundreds of neuronal genes^11^. Strong positive correlation exists between induction of ASCL1 expression and acquisition of NE markers in prostate cancer following androgen deprivation^9,12^, whilst prostate cancer NE transdifferentiation is facilitated by loss of REST^13^. ASCL1 and REST expression appear to be controlled by androgens^9,13^, therefore, may be pivotal in the acquisition of the NE phenotype following ADT.

How expression of key regulators of neurogenesis, ASCL1 and REST are co-ordinated following ADT and iADT, and whether the molecular events associated with transition to the NE-like phenotype are fixed or plastic, are not fully understood. Better definition of the key molecular mechanisms driving NE transdifferentiation is essential to facilitate stratification of CRPC tumours and identify targets for more effective NEPC management. Fully resolving the molecular basis of NEPC lineage plasticity is essential to understand tumour progression, improve the management and sensitization of NEPC to therapeutic strategies, and improve clinical outcomes. Here, the molecular events accompanying NE transdifferentiation of prostate cancer, and their plasticity were investigated using an *in vitro* model of androgen deprivation. Marked nuclear accumulation of ASCL1/hASH1 accompanied NE transdifferentiation of LNCaP cells, and hASH1 localisation persisted, even when the NE-like cells had apparently dedifferentiated back to an epithelial-like phenotype. Here we show, for the first time, that intermittent androgen deprivation and loss of AR signalling may promote the existence of “hybrid” prostate cancer cells that retain both NE-like and epithelial qualities, most notably persistent nuclear localisation of hASH1. As a potent driver of neurogenesis, and clinical marker of NEPC^9^, persistent hASH1 localisation could maintain expression of the transcriptional programs that give rise to NEPC therapeutic resistance and potentially initiate more rapid NE transdifferentiation upon subsequent AD, suggesting iADT may promote aggressive NEPC evolution.

## Results

### Androgen deprivation triggers neuronal-like morphology in androgen sensitive cells

**Figure 1:**
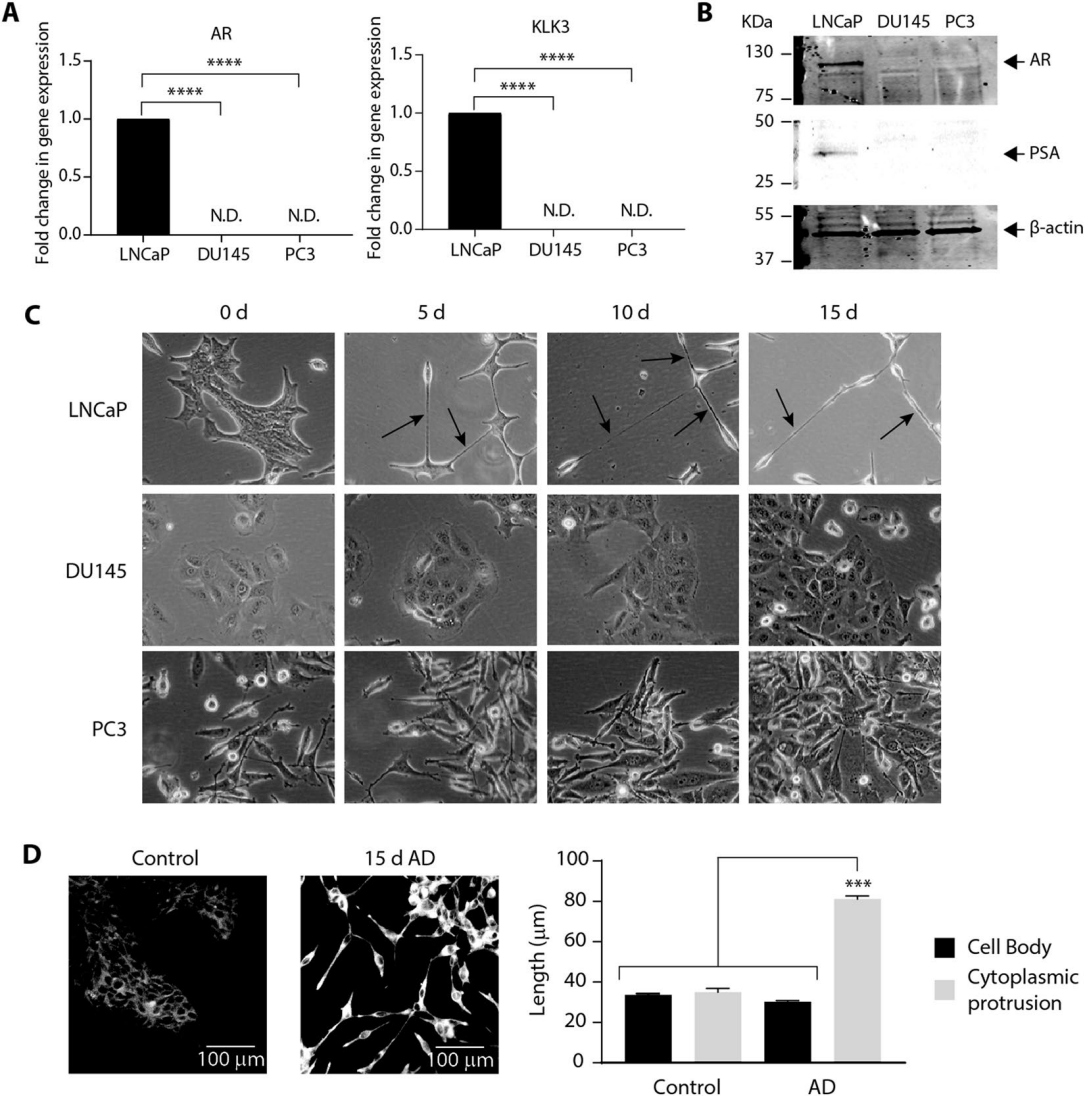
Androgen deprivation triggers significant phenotypic changes in LNCaP cells. **(A**) Relative *Androgen Receptor* (*AR*) and *Kallikrein Related Peptidase 3* (*KLK3*) expression was analysed in LNCaP, DU145 and PC3 prostate cancer cells by qRT-PCR. Gene expression was normalised to β-actin and expressed relative to LNCaP. Data is presented as mean ± SEM and analysed by one-way ANOVA with Tukey’s multiple comparisons; ****p < 0.0001; n = 3; N.D: not detected. (**B)** Representative immunoblot analysis showing AR and Prostate Specific Antigen (PSA) expression in LNCaP, DU145 and PC3 prostate cancer cells. Molecular weights are indicated and equal protein loading was assessed by immunoblotting for β-actin. All uncropped immunoblot images are included in the Supplementary File. (**C)** Representative bright field microscopy images of LNCaP, DU-145 and PC-3 cells cultured for 0, 5, 10 or 15 days (d) in androgen-deprived (AD) conditions (20× magnification). Arrows indicate the presence of neurite-like extensions. (**D**) Representative confocal microscopy images of control or 15d AD LNCaP cells stained for the presence of neurites (20× magnification and 5 × 5 tilescan). Scale bar represents 100 µm. The length of the cell body and cytoplasmic protrusions from at least 100 control and AD LNCaP cells were measured across three visual fields and expressed as mean ± SEM (n = 3) and analysed by one-way ANOVA with Tukey’s multiple comparisons; ***p < 0.001.

The molecular mechanisms involved in transdifferentiation of prostate adenocarcinoma were investigated by culturing androgen sensitive, LNCaP cells and androgen-insensitive, DU145 and PC3^14^ cells (Fig. 1A,B) in phenol-red free medium containing charcoal stripped serum to remove androgens and mimic androgen deprivation (AD)^15,16^. Under control (unstripped serum) conditions, LNCaP, DU145 and PC3 cells displayed an epithelial-like morphology, with PC3 cells often displaying cytoplasmic protrusions (Fig. 1C). LNCaP cells developed short cytoplasmic protrusions by day 5 of AD that became more extensive by 10 and 15 days AD, with cells adopting an elongated, neuronal-like morphology (Fig. 1C). DU145 and PC3 cells did not show any observable morphological changes in AD and after 15 days AD, resembled control cells at day 0 (Fig. 1C).

To facilitate visualisation of the neuronal-like morphology and quantification of protrusion length, LNCaP cells were stained with a fluorescent cell membrane dye^17^ (Fig. 1D). In control LNCaP cells, the cell body and any cytoplasmic protrusions were similar in length (33.7 ± 0.60 µm vs 35.0 ± 1.89 µm respectively), whilst in AD LNCaP cells, protrusions grew to ~2.6 times the cell body length (81.1 ± 1.62 µm vs 30.2 ± 0.67 µm; p < 0.001; Fig. 1D), confirming these AD conditions promote neurite-like formation^18^ and neuroendocrine (NE)-like transdifferentiation of LNCaP cells^19^.

### Androgen deprivation induces expression of the canonical neurogenesis regulator, ASCL1

**Figure 2:**
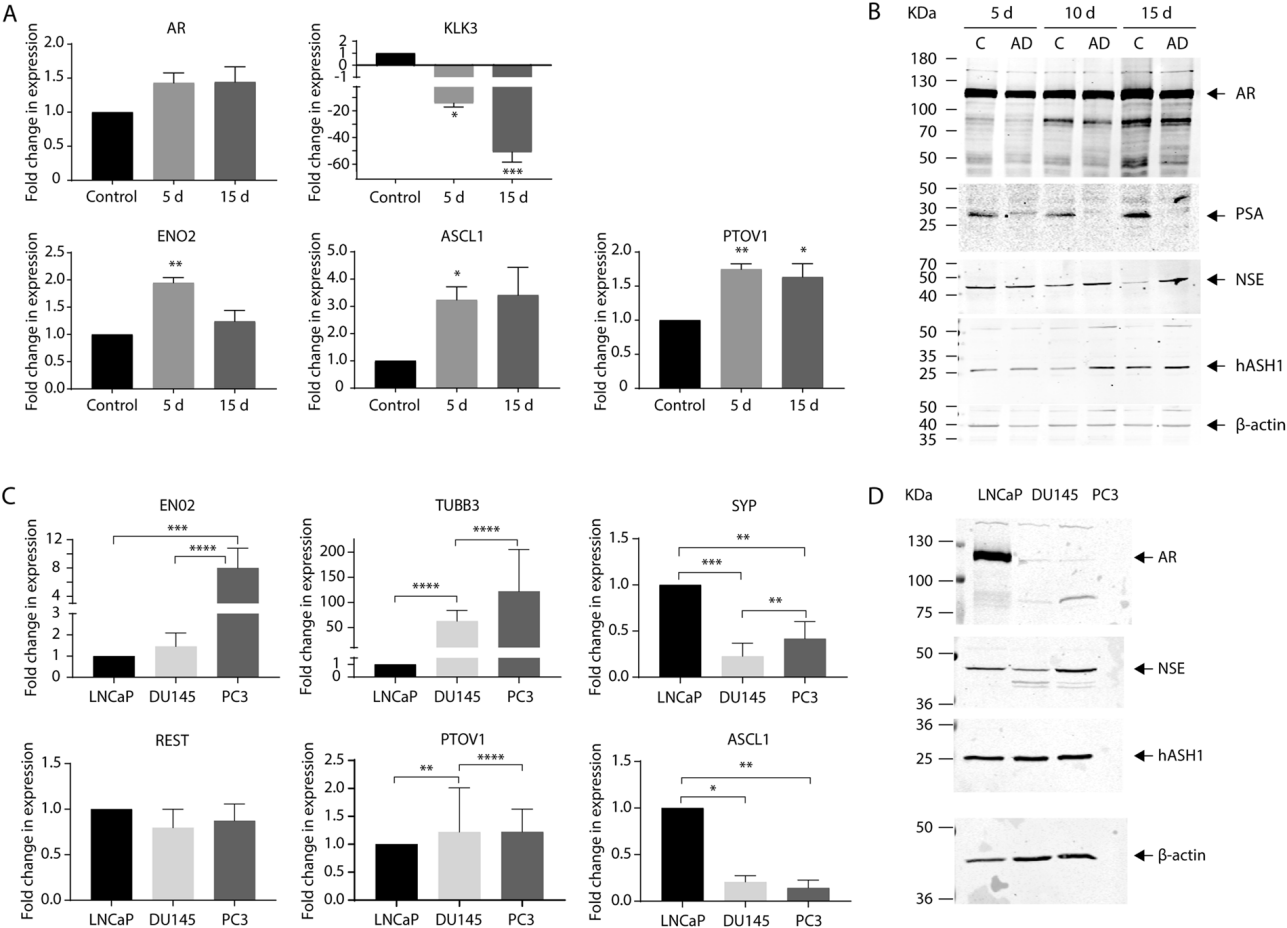
Androgen deprivation upregulates expression of key markers within the canonical neurogenesis pathway. **(A**) Relative expression of *AR*, *KLK3*, *ENO2*, *ASCL1*, *PTOV1* was analysed in control, or androgen deprived (AD; 5 or 15d) LNCaP cells via qRT-PCR. Data is expressed as the mean ± SEM (n = 3) and was analysed by one-way ANOVA with Dunnett’s multiple comparisons; *p < 0.05, **p < 0.01, ***p < 0.001. (**B**) Representative immunoblot analysis showing AR, PSA, NSE and hASH1 expression in LNCaP cells after 5, 10 or 15d growth in control or AD culture conditions. Molecular weights are indicated and equal protein loading was assessed by immunoblotting for β-actin. (**C**) *Neuron-specific enolase* (*ENO2*), *Tubulin Beta 3 Class III* (*TUBB3*), *synaptophysin* (*SYP*), *RE1 Silencing Transcription Factor* (*REST*), *PTOV1 and Achaete-Scute Family BHLH Transcription Factor 1* (*ASCL1*) expression was analysed in LNCaP, DU145 and PC3 prostate cancer cells by qRT-PCR. Gene expression was normalised to β-actin and expressed relative to LNCaP. Data is presented as mean ± SEM (n = 3) and analysed by one-way ANOVA with Tukey’s multiple comparisons; *p < 0.05; **p < 0.01; ***p < 0.001; ****p < 0.0001. (**D**) Representative immunoblot analysis showing AR, NSE and hASH1 expression in LNCaP, DU145 and PC3 in control conditions. Molecular weights are indicated and equal protein loading was assessed by immunoblotting for β-actin. All uncropped immunoblot images are included in the Supplementary File.

The molecular changes underpinning the shift to a neuronal-like morphology in LNCaP cells following AD were assessed by qRT-PCR and immunoblotting looking for key markers associated with androgen signalling, mature neurons and cell fate. AD significantly dampened AR signalling; expression of AR target gene, kallikrein-3 (KLK3, encoding PSA)^20^ was significantly downregulated (p = 0.03; Fig. 2A), and PSA expression was undetectable after 15 days AD (Fig. 2B), whilst AR mRNA and protein expression remained evident throughout (Fig. 2A,B). AD significantly increased expression of cell fate regulator, ASCL1 (p = 0.03) and neuron-specific enolase (ENO2) (p = 0.0036) at both the transcript and protein level (Fig. 2A,B). PTOV1 is a molecular marker for prostate cancer progression^21^ and expression dramatically increases with tumour grade^22^ and prevalence of NE cells^23^. PTOV1 expression was significantly increased by AD (p = 0.0086) and remained elevated 15 days after AD (p = 0.018) (Fig. 2A). Increased expression of key markers of neural fate, mature neurons and NE cells, together with the observed morphological changes (Fig. 1C,D), clearly confirms AD-mediated NE transdifferentiation of LNCaP cells in this model.

Basal expression of neuronal (ENO2/NSE, TUBB3), cell fate (REST, ASCL1/hASH1) and NE (PTOV1/SYP) markers was also assessed in PC3 and DU145 cells (Fig. 2C,D). Marked expression of ENO2/NSE and neuronal beta tubulin (TUBB3) was found in the androgen-insensitive cells compared to LNCaP, with significantly greater expression of ENO2 and NSE in PC3 cells (p < 0.0001) (Fig. 2C,D). There was no significant difference in REST expression between LNCaP, DU145 and PC3 cells, however ASCL1 expression was significantly lower in DU145 and PC3 cells compared to LNCaP cells (p = 0.0124 and 0.035 respectively) (Fig. 2C). Expression of synaptophysin (SYP), a clinical marker of NEPC^24^, was also significantly lower in the androgen-insensitive cells (p = 0.0009 and 0.044 respectively) though expression in PC3 cells was greater than DU145 cells (p = 0.038, Fig. 2C). Curiously, total hASH1 protein expression appeared comparable in all three prostate cancer lines (Fig. 2D).

### Neuroendocrine transdifferentiation of LNCaP cells is associated with nuclear localisation of hASH1

**Figure 3:**
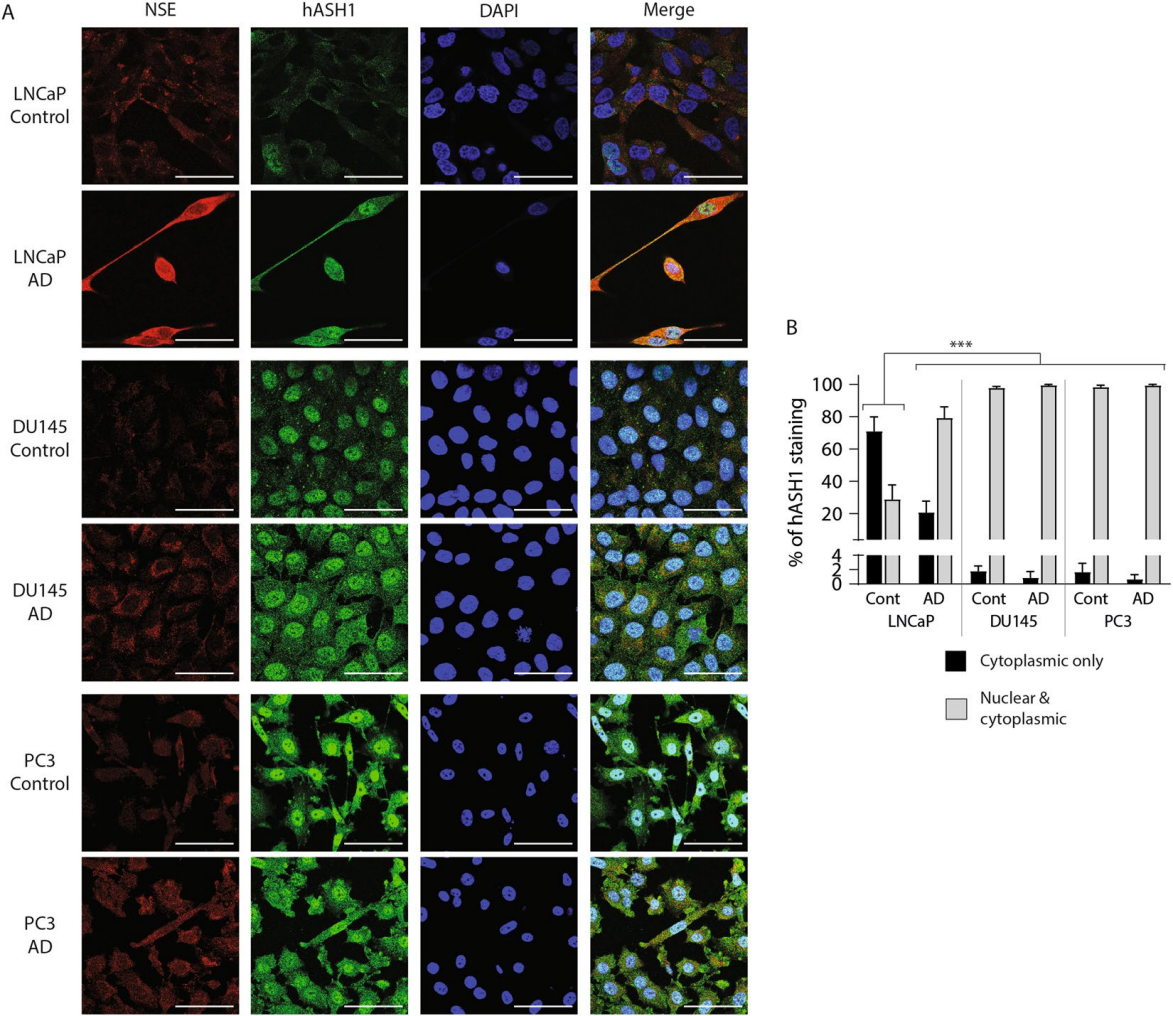
Androgen deprivation and neuronal-like changes in LNCaP morphology are associated with nuclear localisation of hASH1. (**A**) Representative confocal microscopy images of LNCaP, DU145 and PC3 cells grown under control or androgen deprived (AD) conditions (LNCaP: 15 days AD; DU145 and PC3: 10 days AD) and stained with anti-NSE, anti-hASH1 or DAPI (63× magnification) (n > 3). Scale bar represents 50 µm. (**B**) hASH1 localisation in panel A was examined in control or AD LNCaP, DU145 and PC3, counting cytoplasmic only, or nuclear and cytoplasmic hASH1 localisation in at least 100 cells. Data is expressed as mean ± SEM (n > 3) and was analysed by two-way ANOVA with Tukey’s multiple comparisons; ***p < 0.001.

Nuclear localisation permits hASH1 mediated transcription of genes primarily involved in neurogenesis^25^ and nuclear hASH1 staining is evident in NEPC tumour sections via immunohistochemistry^9^, therefore hASH localisation in AD prostate cancer cells was investigated. Diffuse cytoplasmic staining and prominent nuclear exclusion of hASH1 was clearly visible in control LNCaP cells (Fig. 3A,B). Total hASH1 staining markedly increased following AD and was accompanied by a significant increase in perinuclear and nuclear hASH1 staining compared to controls (Fig. 3A,B; p < 0.001). hASH1 staining in DU145 and PC3 cells was visibly greater than LNCaP cells, characterised by diffuse cytoplasmic and prominent nuclear staining. Nuclear hASH1 staining was most evident in PC3 cells where ~ 100% of cells presented nuclear staining (Fig. 3A,B). Nuclear hASH1 localisation was unchanged in DU145 and PC3 cells following AD, but cytoplasmic staining became more pronounced, therefore only LNCaP cells showed any change in localisation of hASH1 upon AD (Fig. 3B). Discrete cytoplasmic NSE staining was observed in LNCaP, DU145 and PC3 cells under control conditions that became more profuse and intense following AD (Fig. 3A), with greatest NSE staining evident in AD LNCaP cells (Fig. 3A). This data indicates hASH1 expression and localisation in prostate cancer cells is sensitive to AR signalling, and AD, or lack of AR signalling, promotes the nuclear localisation of hASH1. Pronounced hASH1 nuclear localisation accompanies the NE transdifferentiation of LNCaP cells, indicating the switch in hASH1 localisation may be a critical step in NE transdifferentiation.

### Androgen supplementation prevents morphological and molecular changes associated with neuroendocrine transdifferentiation

**Figure 4:**
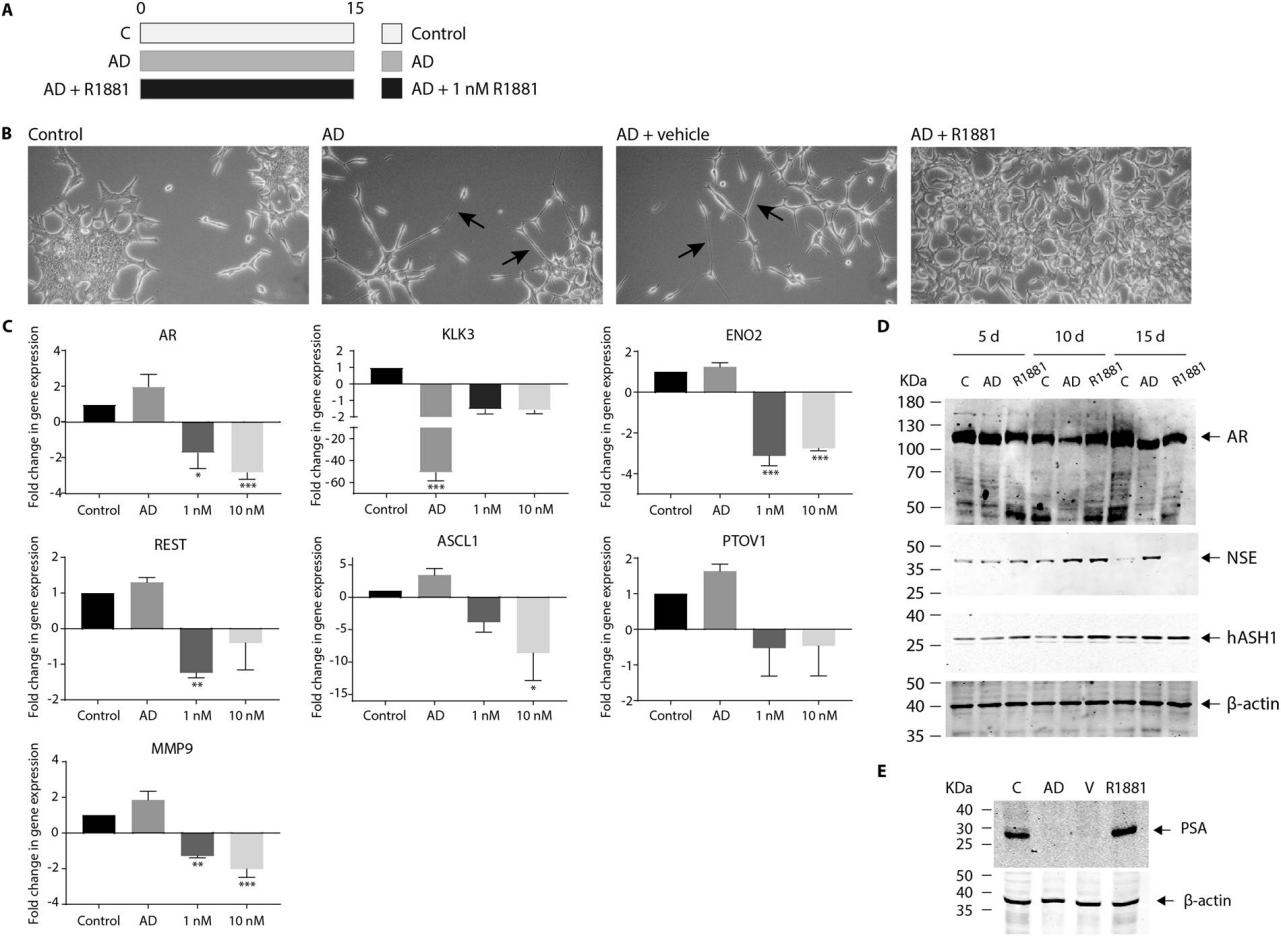
Androgen prevents the acquisition of morphological and molecular changes associated with neuronal-like transdifferentiation of LNCaP cells. (**A**) Schematic diagram indicating the experimental design of androgen manipulation used in these experiments. (**B**) Representative bright field microscopy images of LNCaP morphology following 15 d culture in either control, androgen deprived (AD), AD plus 0.01% DMSO (AD + vehicle) or 1 nM R1881 synthetic androgen (R1881; AD + R1881) conditions (20× magnification). Arrows indicate the presence of neurite-like extensions. (**C**) Relative expression of *AR*, *KLK3*, *ENO2*, *REST*, *ASCL1*, *PTOV1 and MMP9* was analysed in control, or androgen deprived LNCaP cells via qRT-PCR. Cells were AD for 15 d and supplemented with either vehicle (AD), 1 or 10 nM R1881. Data is expressed as the mean ± SEM (n = 3) and was analysed by one-way ANOVA with Dunnett’s multiple comparisons; *p < 0.05, **p < 0.01, ***p < 0.001. (**D**) Representative immunoblot analysis showing AR, NSE and hASH1 expression in LNCaP cells after 5, 10 or 15d growth in control, androgen deprived (AD) or AD plus 1 nM R1881 culture conditions. (**E**) Representative immunoblot analysis showing PSA expression in LNCaP cells after 15d growth in control, androgen deprived (AD) or AD plus 0.01% DMSO (V) or 1 nM R1881 (R1881) culture conditions. (**D**,**E**) Molecular weights are indicated and equal protein loading was assessed by immunoblotting for β-actin. All uncropped immunoblot images are included in the supplementary file.

Charcoal stripping removes several lipophilic molecules from growth serum, including steroid hormones such as androgens^16^. To confirm the morphological and molecular changes observed following charcoal stripping were exclusively due to lack of androgen, LNCaP were cultured in charcoal-stripped media supplemented with synthetic androgen (R1881; Metribolone; Fig. 4A), employing concentrations used by others^26^. The long neurite-like extensions evident in AD LNCaP cells (Fig. 4B) failed to develop in cells cultured in AD plus R1881, and morphology was indistinguishable from cells grown in control, unstripped media (Fig. 4B). Vehicle (DMSO) had no impact on neurite formation in AD LNCaP cells (Fig. 4B) confirming the morphological features triggered by charcoal stripping serum are due to a lack of androgen (Fig. 4B).

R1881 supplementation significantly decreased AR mRNA expression in AD LNCaP cells (Fig. 4C; p = 0.01 and p < 0.001 respectively), however, little change in AR protein expression was evident (Fig. 4D). The AD-dependent silencing of AR signalling was blunted by addition of R1881, and KLK3 and PSA expression in R1881 treated AD cells remained robust (Fig. 4C, p < 0.001; Fig. 4E). R1881 treatment also prevented the AD-dependant increase in expression of markers of neuronal fate (REST, ASCL1) and neuronal differentiation (NSE). Significant reductions in ENO2 (p < 0.001), REST (p = 0.0092), and ASCL1 (p = 0.0446) expression were clearly evident in the presence of R1881 (Fig. 4C) and by 15 days NSE expression was undetectable (Fig. 4D). The AD-dependent increase in PTOV1 expression was also inhibited by R1881, although not significantly (Fig. 4C). Therefore, activation of canonical neuronal fate pathways and NE transdifferentiation in AD LNCaP cells is androgen dependent. MMP9 is associated with aggressive prostate tumour growth^27^. MMP9 expression was not dramatically altered by AD, yet was significantly decreased by R1881 treatment (Fig. 4C; p = 0.0037 and 0.0006 respectively). This hints that maintaining androgen concentrations may restrict MMP-9 expression and the invasive potential of prostate cancer epithelium.

### Exposure to androgen reverses established NE-like transdifferentiation in AD LNCaP cells

**Figure 5:**
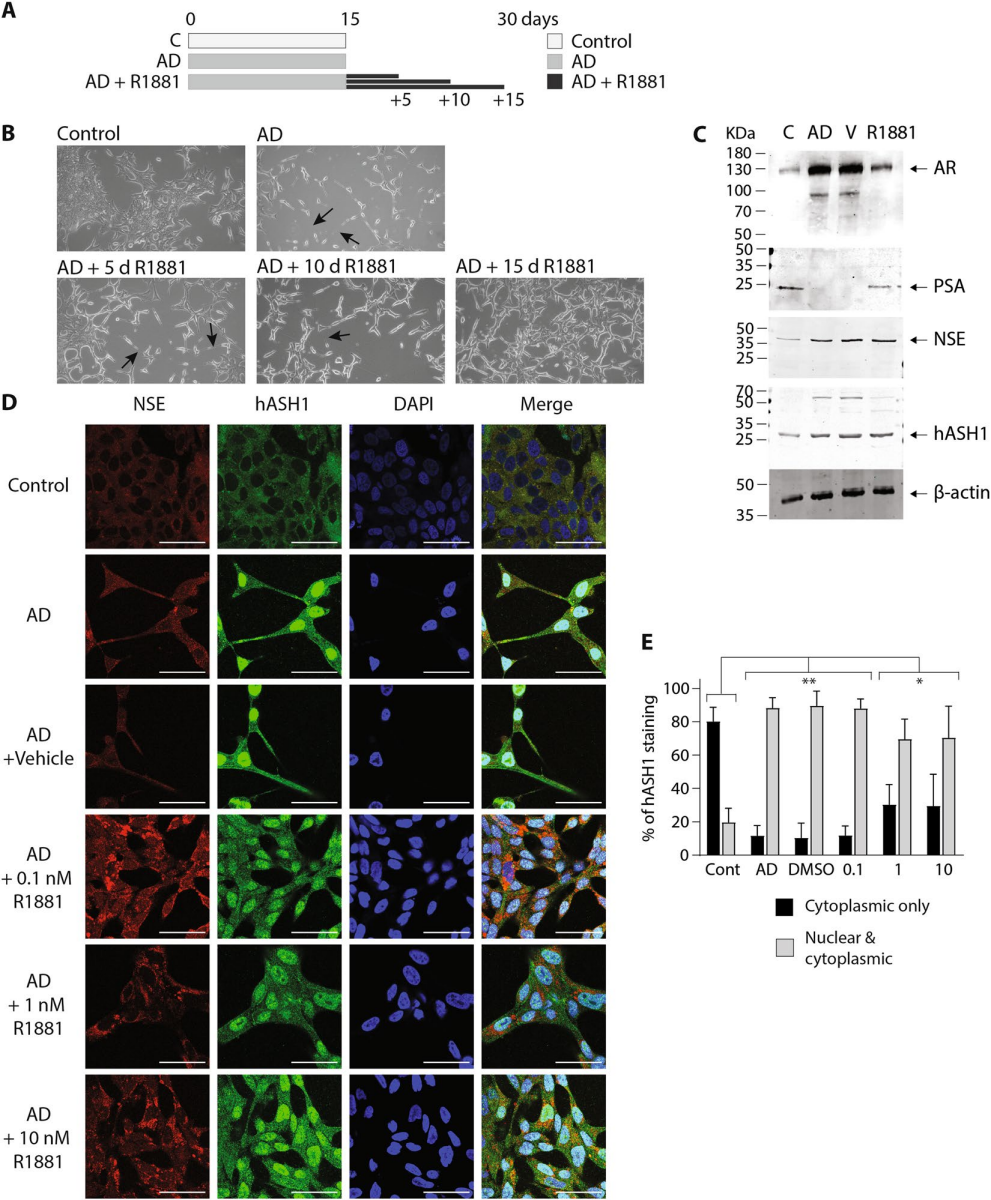
Reintroduction of androgen reverses the NE-like morphology in prostate cancer cells. (**A**) Schematic diagram indicating the experimental design of androgen manipulation used in these experiments, showing the number of days LNCaP cells were cultured in the control, androgen deprived (AD) or androgen deprived plus R1881 conditions. (**B**) Representative bright field microscopy (20x magnification) images of LNCaP cells grown under control and androgen deprived (AD) compared to AD LNCaP cells cultured for a further 5, 10 or 15d following AD in the presence of 1 nM R1881. The presence of neurite-like extensions are indicated by arrows. (**C)** Representative immunoblot analysis showing AR, PSA, NSE and hASH1 expression in LNCaP cells grown in control or AD conditions, or AD followed by 15 days of AD conditions plus DMSO vehicle (V) or 1 nM R1881. Molecular weights are indicated and equal protein loading was assessed by immunoblotting for β-actin. All uncropped immunoblot images are included in the Supplementary File. (**D**) Representative individual and merged confocal microscopy images (63× magnification) of LNCaP cells stained with anti-NSE, anti-hASH1 or DAPI in control, androgen deprived (AD) or AD LNCaP cells cultured for a further 15d in the presence of 0.1% DMSO; (AD + vehicle) or 0.1, 1 or 10 nM R1881 (AD + R1881). Scale bar represents 50 µm. (**E**) hASH1 localisation in panel D was analysed, counting cytoplasmic only, or nuclear and cytoplasmic hASH1 localisation in at least 100 cells. Data is expressed as mean ± SEM (n = 3) then analysed by two-way ANOVA with Tukey’s multiple comparisons; *p < 0.05, **p < 0.01.

The plasticity of molecular changes involved in NE transdifferentiation was assessed by culturing LNCaP cells in AD conditions for 15 days to develop the NE-like phenotype, followed by a further 15 days in control, androgen rich conditions (AD media plus R1881; Fig. 5A). The long neurite-like extensions produced by AD of LNCaP cells were rapidly lost following reintroduction of R1881 (Fig. 5B) and 15 days post-AD, LNCaP morphology resembled control cells (Fig. 5B,D). This further emphasises Shen *et al*.^8^ findings that morphological changes induced by AD are plastic.

Transdifferentiated NE-like cells are thought to be hormone refractory and resistant to ADT, due to lack of AR expression^3,28^. AR target gene, PSA^20^ expression was evident in control (unstripped serum) conditions, yet undetected following AD, indicating loss of canonical AR signalling (Fig. 5C); reintroduction of R1881 fully restored PSA expression in AD LNCaP cells (Fig. 5C). Full-length AR expression increased under AD conditions and was accompanied by a smaller AR reactive band at ~90 kDa (Fig. 5C). This was lost upon reintroduction of R1881, whilst full-length AR expression returned to control levels (Fig. 5C). NE-like LNCaP cells, therefore retain AR expression, androgen sensitivity and the capacity for canonical AR gene transcription, even after periods of AD.

Interestingly, reintroduction of androgen did not reverse all of the molecular changes associated with NE-like transdifferentiation of LNCaP cells. Notably, NSE and hASH1 expression remained elevated post-AD and R1881 treatment and was comparable to AD cells (Fig. 5C). Whole cell staining showed robust nuclear and perinuclear hASH1 staining in AD cells treated with R1881 (Fig. 5D,E), even at higher concentrations of R1881 (10 nM), and the prominent nuclear exclusion of hASH1 seen in control cells, was not observed. NSE and hASH1 expression was unaffected by the presence of vehicle (Fig. 5D,E). Quantification of hASH1 localisation suggests a slight concentration-dependent reduction in nuclear hASH1 staining at higher R1881 concentrations (Fig. 5E) further supporting the role of AR in regulating hASH1 localisation. Key molecular features of NE transdifferentiation are therefore retained in LNCaP cells post-AD, even following reintroduction of androgen. Induction and nuclear localisation of hASH1 in LNCaP cells may represent a more permanent adaption to androgen deprivation.

### The impact of intermittent AD on NE-like transdifferentiation of LNCaP cells

**Figure 6:**
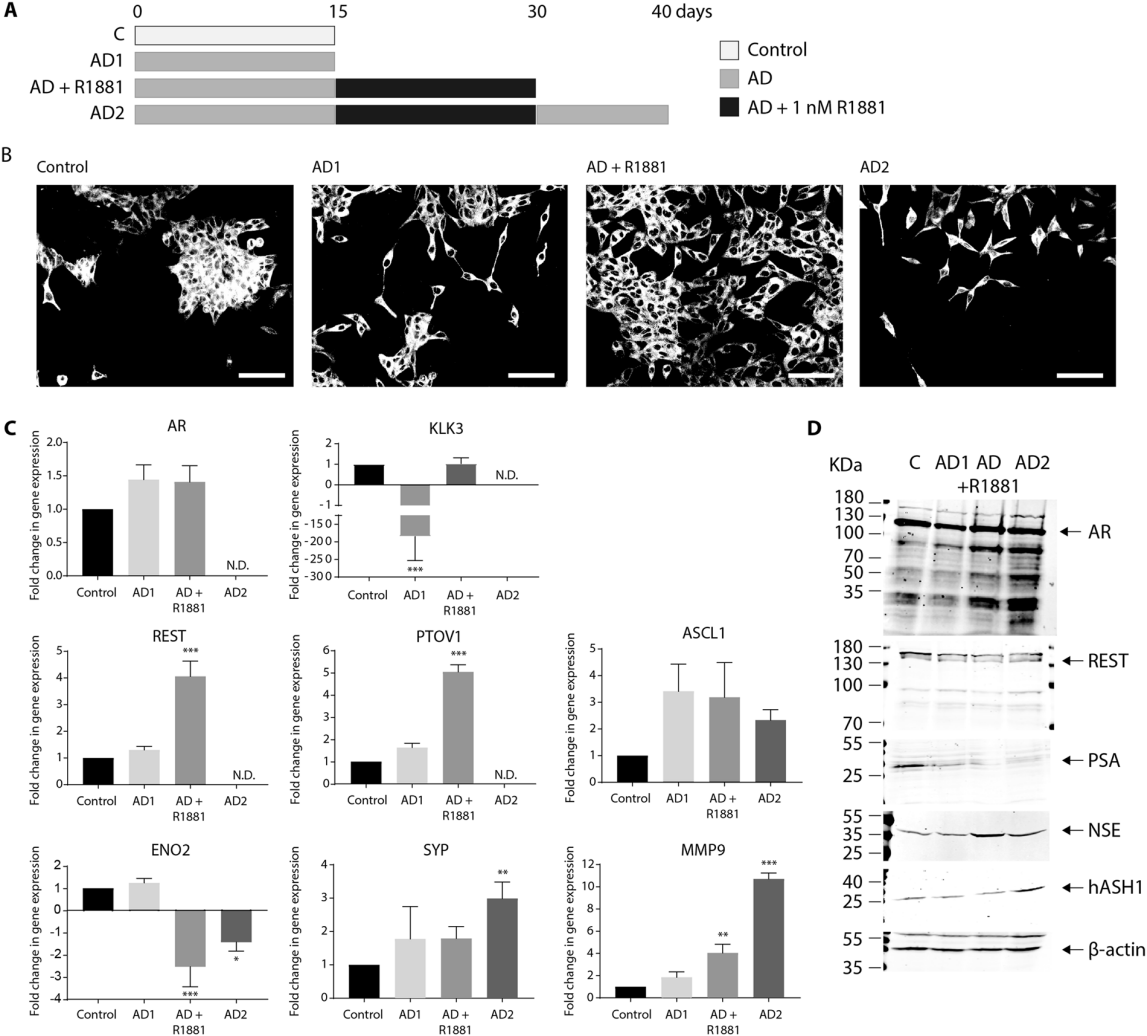
The impact of intermittent AD on NE transdifferentiation of LNCaP cells. (**A**) Schematic diagram indicating the experimental design used to generate intermittent AD (iAD) showing the number of days LNCaP cells were cultured in the control, androgen deprived (AD) or androgen deprived plus R1881 conditions. (**B**) Representative confocal microscopy images of LnCaP cells grown under control AD or iAD condition and stained for the presence of neurites (20× magnification). AD1: 15d AD; AD + R1881: 15d AD followed by 15d in AD media containing 1 nM R1881; AD2: 15d AD followed by 15d AD + R1881, then 10d in AD conditions. Scale bar represents 100 µm. (**C**) Relative expression of *AR*, *KLK3*, *REST*, *PTOV1*, *ASCL1*, *ENO2*, *SYP and MMP9* was analysed in control, AD1, AD + R1881 or AD2 treated LNCaP cells via qRT-PCR. Data is expressed as the mean ± SEM (>n = 3) and was analysed by one-way ANOVA with Dunnett’s multiple comparisons; *p < 0.05, **p < 0.01, ***p < 0.001. N.D.: not detected. (**D**) Representative immunoblot analysis showing AR, REST, PSA, NSE and hASH1 expression in LNCaP cells grown in control, AD1, AD + R1881 or AD2 conditions. Molecular weights are indicated and equal protein loading was assessed by immunoblotting for β-actin. All uncropped immunoblot images are included in the Supplementary File.

Prostate cancer patients are often given intermittent androgen deprivation therapy (iADT) to minimise the adverse effects of ADT^7^. Herein, iADT was modelled, *in vitro*, by culturing LNCaP cells in sequential cycles of AD (AD1 and AD2) interspersed with growth in androgen rich conditions (AD + R1881; Fig. 6A). Neurite-like projections triggered by the initial cycle of androgen deprivation (AD1) were rapidly lost by addition of R1881 (AD + R1881; Fig. 6B), however, LNCaP cells re-acquired neurite-like projections upon exposure to a second cycle of AD (AD2), and morphology following exposure to AD2 was comparable to LNCaP cells after AD1 (Fig. 6B). This reversible morphological change in response to fluctuating androgens highlights LNCaP plasticity and the malleability of the NE-like phenotype (Fig. 6B)^8^. AR expression was slightly elevated following AD1, and remained elevated post-AD following reintroduction of R1881 (AD + R1881; Fig. 6C). Robust KLK3 expression was significantly reduced by AD (*p* = < 0.001), yet returned to basal levels after R1881 supplementation (Fig. 6C). LNCaP cells therefore maintain AR signalling capacity during transdifferentiation into NE-like cells, and re-establish aspects of canonical AR signalling following iAD.

Curiously, AR mRNA was undetectable following AD2 with concurrent loss of detectable KLK3 expression (Fig. 6C). By contrast, full-length AR protein expression was maintained even in the absence of androgen (AD1 and AD2), and was accompanied by several smaller AR responsive bands (Fig. 6D; ~90, ~55 and ~30 kDa). These are most likely AR degradation products as the antibody used for immunoblotting recognises an epitope in the AR’s NH_2_ terminal region. AD1 reduced PSA expression compared to control cells, and PSA expression was further reduced by the second cycle of AD (AD2; Fig. 6D).

### Intermittent AD primes LNCaP cells for NE-like transdifferentiation

The impact of iAD on drivers of neuronal cell fate was also assessed. Basal REST and PTOV1 expression was not altered by AD, yet post-AD, REST and PTOV1 expression was significantly increased following R1881-mediated reversal of the NE-like phenotype (*p* = < 0.001, Fig. 6C). Further AD led to undetectable expression of both REST and PTOV1. This is in keeping with the return of LNCaP cells to neuronal-like morphology, the role of REST as an inhibitor of neurogenesis^11^, and the hypothesis that PTOV1 may be involved in disrupting cell fate^29,30^. By contrast, basal ASCL1 expression remained persistently elevated post-AD upon reintroduction of R1881 (Fig. 6C) and was further elevated by a subsequent round of AD (AD2, Fig. 6D). Together, this indicates the AD-dependant increase in hASH1 expression is resistant to androgen (Figs. 2 and 4) and iAD can trigger incremental increases in hASH1 expression.

Expression of the neuronal marker, ENO2 was not increased by AD, yet was significantly reduced post-AD by reintroduction of androgen (Fig. 6C, *p* = < 0.001), in keeping with the observed loss of neuronal-like morphology in R1881 treated cells (Fig. 6B). Exposure to AD2 increased ENO2 expression in LNCaP cells compared to AD + R1881 treated cells, yet remained significantly below control LNCaP and AD1 expression (Fig. 6C; *p* = 0.01). By contrast, iAD promotes NSE protein expression, and expression was further elevated by AD2 (Fig. 6D) indicating an increased NE-like phenotype. Synaptophysin (SYP) was also increased by AD treatment. Like ASCL1, SYP expression remained elevated post-AD when NE morphology was reversed (Fig. 6C) and was further increased upon a subsequent cycle of AD (*p* = 0.002; Fig. 6C). MMP-9 expression correlates with CRPC progression and increased prostate cancer aggression^27^. MMP9 expression was increased in an incremental fashion by sequential rounds of AD in LNCaP cells (*p* = 0.001 and p < 0.001 respectively; Fig. 6C) and similar to ASCL1, MMP-9 expression remained elevated upon R1881 treatment (Fig. 6C). Together, these findings show key molecular events associated with NE transdifferentiation persist in prostate cancer cells, even following reintroduction of androgen and reversal in morphology. This suggests NE-like cells do not fully revert back to their epithelial-like state in the presence of androgen and may retain certain NE features in a “hybrid” phenotype.

### Nuclear localisation of hASH1 is maintained during intermediate androgen deprivation

**Figure 7:**
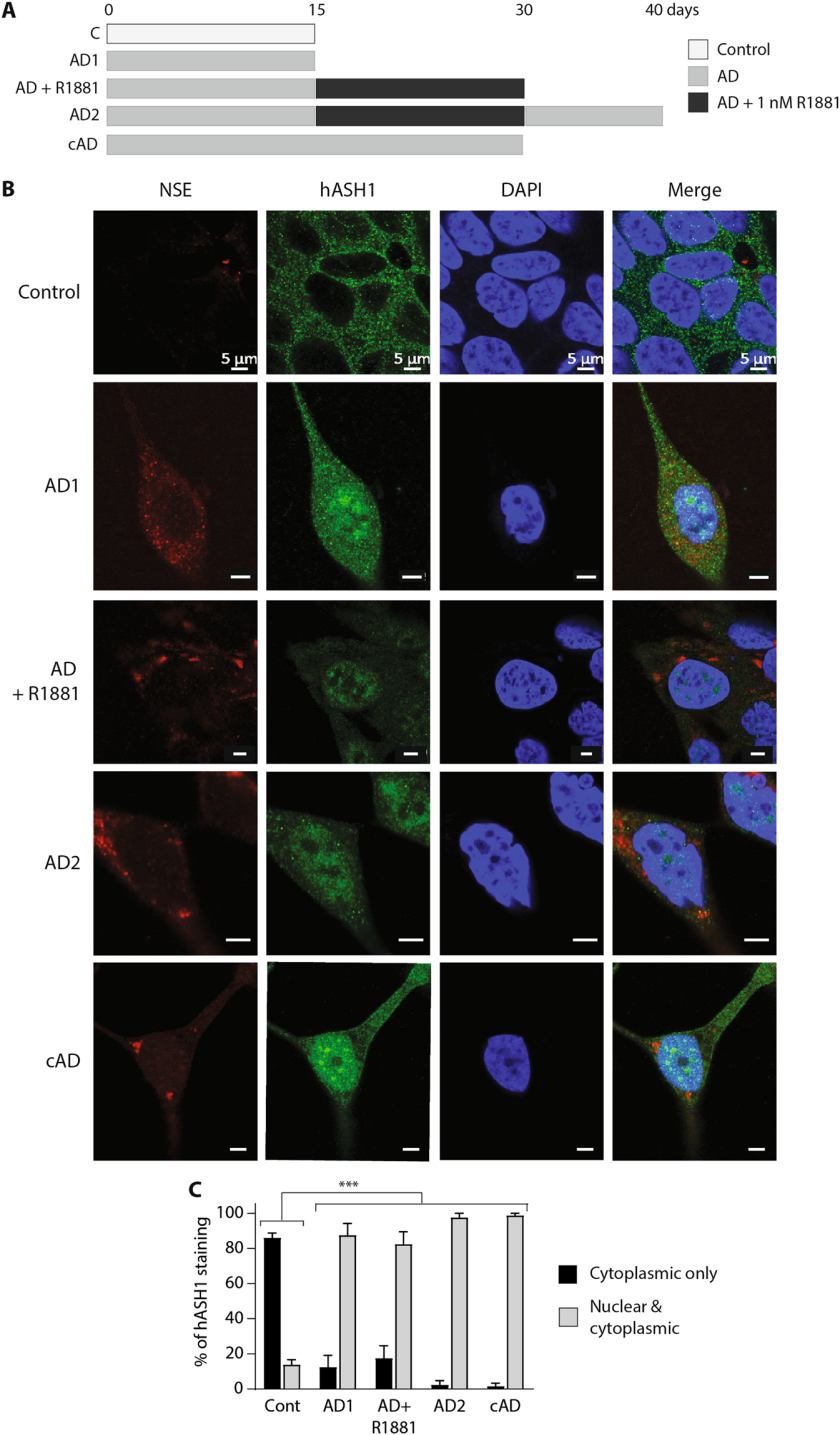
Intermittent AD alters the subcellular localisation of hASH1. (**A)** Schematic diagram indicating the experimental design used to generate intermittent AD or continuous (c) AD showing the number of days LNCaP cells were cultured in the control, androgen deprived (AD) or androgen deprived plus R1881 conditions. (**B**) Representative individual and merged confocal microscopy images of LNCaP cells stained with anti-NSE, anti-hASH1 or DAPI after 15 d culture in control, AD1, AD2, AD + R1881, or cAD conditions. Scale bar represents 5 µm. (**C**) hASH1 localisation in panel B was analysed, counting cytoplasmic only, or nuclear and cytoplasmic hASH1 localisation. Data is expressed as mean ± SEM (n = 3) then analysed by two-way ANOVA with Tukey’s multiple comparisons; ***p < 0.001. (**A**–**C)** C: control; AD1: 15d AD; AD + R1881: 15d AD followed by 15d in AD media containing 1 nM R1881; AD2: 15d AD followed by 15d AD + 1 nM R1881, then 10d in AD conditions; cAD: 30d in AD conditions.

AD triggers an increase in hASH1 nuclear localisation and global staining intensity. Whilst R1881 treatment post-AD reduced hASH1’s overall staining intensity, hASH1 nuclear localisation persisted in LNCaP cells, despite the loss of NE morphology (Fig. 7B, also 5D). hASH1 was further responsive to AD and AD2 triggered incremental increases in nuclear and cytoplasmic hASH1 staining (Fig. 7B,C). NSE staining remained evident post-AD in R1881 treated cells, and became more pronounced and punctate with progressive rounds of AD (Fig. 7B,C). Continuous AD (cAD) produced intense hASH1 nuclear localisation and distinct, punctate NSE staining in LNCaP cells (Fig. 7B,C). Certain molecular features associated with NE transdifferentiation may therefore persist post-AD, even following reintroduction of androgen, suggesting apparently dedifferentiated NE-like cells may exist in a “hybrid” phenotype, with characteristics of both adenocarcinoma and NE-like cells.

## Discussion

Prostate adenocarcinoma rapidly acquires a NE-like phenotype and expresses NE markers under the potent selective pressure of androgen deprivation and AR inhibition (Figs. 1 and 2)^31^, highlighting the intrinsic capacity of the prostate epithelium to transdifferentiate into NE-cells and the central role of AR signalling in maintaining an epithelial phenotype. Aberrant expression of several regulators of neurogenesis, cell fate and lineage commitment is found in prostate cancer. REST is the major negative regulator of neurogenesis, restricting differentiation of neuronal stem cells^11^ whilst ASCL1 positively regulates neural progenitor differentiation and commitment to neuronal lineages^10^. REST expression is lost in NEPC and negatively correlates with neuronal transdifferentiation of prostate cancer^5,13^, whilst a strong positive correlation between ASCL1 expression and acquisition of NE-like cells exists in prostate cancer^9,12^. Temporal and spatial changes in ASCL1 expression also correlate with transdifferentiation of prostate cancer cells (Figs. 2 and 3). Aberrant expression of key regulators of neurogenesis is therefore intricately linked with neuronal transdifferentiation of prostate cancer, expression of NE markers and highly aggressive NEPC growth^5,12,13^.

Transcriptional control of AR target genes is mediated by AR binding to AR response elements (ARE) in genomic promoter regions^32^. AR directly modulates REST transcriptional activity by localising to RE-1 elements in the promoters of REST regulated genes^13^. A marked reduction in REST expression was observed upon R1881 reintroduction to androgen deprived (AD) conditions indicating REST expression is responsive to AR (Figs. 4 and 6). Whether this represents direct ARE-driven regulation of REST expression remains unknown. Fluctuations in ASCL1 expression in response to AR signalling have been suggested, but not proven, at the molecular level^9,12^ and never assessed in iADT. ASCL1 expression was potently induced in LNCaP cells by AD (Fig. 2) whilst synthetic androgen markedly reduced ASCL1 expression below basal levels (Fig. 4), indicating ASCL1 is highly responsive to androgen and may even be transcriptionally regulated by the androgen signalling axis. Interaction or cross-talk between AR and ASCL1 at ARE or ASCL1 consensus sites on key target genes, in a manner similar to REST has, however, not been documented. ASCL1 is a potent oncogene, driving invasive growth and metastasis of many tumours^33^. Considering the oncogenic potential of ASCL1 and marked ASCL1 expression in NEPC, determining whether AR acts upstream of ASCL1 to attenuate ASCL1 expression and prevent NE transdifferentiation is fundamental to our understanding of NEPC evolution. Future work will establish if ASCL1 expression is directly regulated by AR signalling.

hASH1 localisation appears to be sensitive to androgen signalling; AD caused marked hASH1 accumulation and nuclear localisation in LNCaP cells, whilst prominent hASH1 staining and nuclear localisation was found in androgen-insensitive, DU145 and PC3 cells under control, unstripped conditions (Fig. 3). The observed reduction in hASH1 nuclear staining in AD LNCaP cells treated with higher concentrations of R1881 (Fig. 5E) would support this. AR signalling may therefore repress hASH1 expression and negatively regulate hASH1’s localisation. Clear hASH1 induction and nuclear localisation was observed in AD LNCaP cells, yet hASH1 staining was most intense in control PC3 cells (Fig. 3B). Charcoal stripping growth serum depletes, but does not eradicate androgens from the culture medium^16^, therefore, intense hASH1 staining may be more evident in PC3 cells due to a complete lack of AR signalling, and therefore AR-dependent repression of hASH1 in these cells^14^.

hASH1 expression and stability is regulated by ubiquitin-mediated proteasomal degradation^34^, however, chromatin-bound and cytoplasmic hASH1 exhibit distinct ubiquitination profiles. As a result, chromatin-bound hASH1 is more resistant to proteasomal degradation and more stable^35^. Differential hASH1 stability and reduced turnover of chromatin-bound hASH1 may account for persistent hASH1 nuclear accumulation in apparently dedifferentiated NE-like LNCaP cells. hASH1’s half-life is however relatively short (~1 hr)^35^ suggesting additional factors maintain hASH1’s persistent nuclear localisation post-AD (Figs. 5 and 7) or in cells lacking AR signalling (Fig. 3). Androgens indirectly regulate the stability of p53 and REST transcription factors^13^ through transcriptional regulation of E3 ligases involved in their post-translational modification^36^. Determining whether androgens also transcriptionally regulate key enzymes involved in hASH1 stability and turnover is key to fully comprehending the link(s) between androgen signalling, ASCL1 mediated neurogenesis and NEPC development. Such knowledge will reveal novel routes of androgen-dependent repression of the prostate cancer’s intrinsic capacity for NE transdifferentiation, and uncover potentially novel, druggable targets for the blockade of NEPC development.

NE cells have significant oncogenic potential and can provide invaluable support to surrounding tumour cells through paracrine secretion of peptide and hormone growth factors^5^. This combined with NE-like cells’ inherent resistance to ADT^28^ explains why NEPC is not only treatment resistant, but highly aggressive. NE-like cells rapidly lose their NE phenotype upon reintroduction of androgen to the culture medium indicating key molecular aspects of NE transdifferentiation, including REST expression, are not fixed (Fig. 5)^8,9,37^. Surprisingly, not all of the molecular changes driving LNCaP NE transdifferentiation were reversed by R1881 treatment, in spite of the apparent reversibility of NE morphology. Marked hASH1 nuclear translocation and accumulation was observed following AD (Fig. 3), however, relocalisation of hASH1 to the cytoplasm post-AD following reintroduction of R1881 was not evident (Figs. 5 and 7), and hASH1 nuclear expression persisted through successive AD cycles. Therefore, certain NE related changes in gene and protein expression are reversible, whilst others may become fixed. Apparently dedifferentiated prostate cancer cells may, therefore, retain aspects of NE transdifferentiation post-AD, such as persistent hASH1 signalling capacity, and exist in a hybrid state between NE-like and epithelial-like phenotypes. Curiously, PC3 cells also appear to exhibit a mixed phenotype; PC3 have an epithelial morphology yet display pronounced expression of mature neuronal markers, ENO2/NSE and TUBB3 and intense nuclear hASH1 staining (Figs. 2C,D and 3A) in androgen rich conditions. By contrast, expression of Syp is lower in PC3 cells (Figs. 2C vs 6C) suggesting they may be more neuronal-like than NE-like and their “hybrid state” may differ from that of apparently dedifferentiated LNCaP cells. Fully understanding whether NE-like cells truly revert to their original state following androgen reintroduction or whether molecular events associated with NE transdifferentiation are retained in a hybrid phenotype is crucial to the improved understanding of NEPC evolution. Establishing whether persistent hASH1 nuclear localisation in previously AD cells, or androgen-insensitive cells, is accompanied by an active and sustained global program of ASCL1-mediated, pro-neural gene transcription in apparent dedifferentiated adenocarcinoma cells, is also key.

iADT is thought to be beneficial^6,38^, possibly delaying delay prostate adenocarcinoma progression to CRPC by restoring apoptotic potential and sensitivity to treatment^39^. Whether iADT causes NE-like cells in the tumour to revert to adenocarcinoma, is unknown. However, the observation that expression of hASH1, certain markers of NE cells (NSE/SYP) and invasive tumour potential (MMP-9) remain elevated in apparently dedifferentiated prostate cancer cells has implications for the efficacy of intermittent ADT (iADT), as it suggests these cells have greater aggressive potential and could more readily undergo NE transdifferentiation upon subsequent AD. iADT could potentially prime prostate adenocarcinoma for more rapid transdifferentiation into NE-like cells upon subsequent ADT cycles and drive aggressive CRPC development. The impact of prostate cancer transitioning between a NE-like and epithelial phenotype, and aggressive tumour growth has yet to be established; in particular, whether iADT therapy accelerates NEPC evolution once CRPC has formed, or facilitates tumour reseeding. Fully appreciating the impact of iAD and androgen fluctuation on NEPC evolution is essential to determine whether treatment breaks protect or predispose patients to more rapid NEPC formation, and whether continuous AD may be preferable to stall the inevitable acquisition of NEPC.

NE transdifferentiation occurs in response to a diverse array of stressful stimuli, including intrinsic tumour hypoxia^40^, inflammation^41^, irradiation^42^, as well as stringent ADT^28^. Androgen-insensitive cells can also transdifferentiate into NE-like cells under conditions, such as forskolin treatment^43^, suggesting prostate adenocarcinoma is hardwired to transdifferentiate into NE-like cells in response to stress. The multitude of stimuli and array of molecular drivers proposed to be involved in NE transdifferentiation indicate the inherent plasticity of prostate adenocarcinoma following stress and convergence of multiple adaptive pathways on NEPC development. Such plasticity may contribute to heterogeneity of CRPC tumours^44^. Better knowledge of these pathways, their hierarchy, temporal impact and importance in NE transdifferentiation is essential to identify new therapeutic targets to stall NE transdifferentiation of CRPC to the lethal NEPC form.

NE cells are not restricted to the prostate; tumours of the lung, thyroid, GI tract and pancreas also contain increased prevalence of NE-like cells^45^. Mutations in the MEN1, VHL and NF1 pathways drive development of sporadic primary NE tumours in the pancreas and GI tract^46^, whilst ASCL1 is pivotal to development and survival of aggressive NE lung tumours^47,48^. The importance of ASCL1 in NE tumour survival does, however, highlight the potential vulnerability of tumours heavily dependent on ASCL1. Indeed, downstream targets of ASCL1 signalling have been suggested as viable drug targets to restrict NE non-small cell lung cancer growth^47^. With the prevalence of NEPC set to increase due to use of ever more potent inhibitors of androgen signalling, (such as enzalutamide and abiraterone^49^), it is imperative we gain a fuller understanding of the molecular mechanisms driving NEPC formation to design more effective therapeutics to this lethal tumour subtype. The potential druggability of the ASCL1 axis combined with increased understanding of the role of AR and ASCL1 in NEPC progression may open exciting new avenues for future drug development and therapeutic options for NEPC. It will also permit more active targeting of potentially dormant NE-like cells primed by iADT to restrict aggressive prostate cancer growth. Players within the ASCL1 axis could also be used as key molecular markers to indicate NE physiology and the vulnerability of CRPC tumours to developing into NEPC. Given the limited prognosis of NEPC, this knowledge will inform strategies to develop targeted therapies towards NEPC subtypes with the aim of significantly improving the management and treatment of NEPC.

## Methodology

### Cell lines and culture conditions

LNCaP cells were obtained from the American Tissue Culture Collection (ATCC). DU-145 and PC-3 cells were a generous gift from Professor Ross (Tissue Injury and Repair Group, University of Edinburgh, UK). Cells were cultured in RPMI-1640 medium containing 10% (v/v) heat-inactivated foetal bovine serum (FBS, Gibco, UK), 2 mM L-glutamine and 100 units/mL (v/v) penicillin and 100 μg/mL streptomycin (Gibco, UK), at 37 °C in a humidified incubator containing 5% (v/v) CO_2_ and passaged once confluent.

### Androgen deprivation

LNCaP cells were seeded at 1 × 10^6^ cells in T75 flasks and grown to 50% confluence. To simulate androgen deprivation (AD), medium was removed and cells washed with saline before adding phenol-red free RPMI 1640 (Gibco, 11835–063) supplemented with 10% (v/v) heat inactivated, charcoal-stripped FBS (SIGMA, F6765), 2 mM L-glutamine, 100 units/mL (v/v) penicillin and 100 μg/mL streptomycin. Matched flasks of LNCaP cells were grown in control unstripped medium. Cells were cultured for between 5–30 days depending on the experiment, replacing the medium every 3–4 days. AD cells did not become confluent within the 15 day treatment so were not passaged; for control and longer AD experiments, cells were trypsinised when confluent and re-plated in control or AD media.

### Synthetic androgen treatment

Synthetic androgen, R1881 (10 µM, SIGMA)^26^.was prepared in DMSO. Cells were seeded at 1 × 10^6^ in T75 flasks. The next day, media was replaced with AD media containing 0.01% (v/v) DMSO (vehicle control), 1 nM or 10 nM R1881 and cells were grown for a further 15 days. Cells were also subjected to AD for 15 days, then treated with R1881 or vehicle for a further 15 days. Culture medium containing R1881 or DMSO was replaced every 3–4 days. When confluent, cells were trypsinised and re-plated in AD medium containing R1881 or DMSO.

### RNA isolation and cDNA synthesis

Cells were harvested by scraping into ice-cold PBS and collected by centrifugation (5 minutes at 840 × g; 4 °C) in a bench top centrifuge. Total RNA was isolated from cells using TRIsure (Bioline, UK) according to manufacturer’s protocol and resuspended in 0.1% DEPC-treated water. RNA concentration and integrity was assessed via Bioanalyser 2100 (Agilent Technologies, UK); samples with an RNA integrity (RIN) >9 were used in quantitative polymerase chain reactions (qPCR)^50^. Total RNA (2 µg) was reverse transcribed using NanoScript 2 RT Premix kit (PrimerDesign, UK); control reactions lacked RT enzyme (-RT).

### Oligonucleotides

Were obtained from MWG Eurofins (UK). Androgen receptor (*AR*, NM_000044.4): F: 5′-CACTGCTACTCTTCAGCATTATTCC-3′, R: 5′-ATGCAGCTCTCTCGCAATAGG-3′; *ASCL1*, (NM_004316.3): F: 5′-AAGCAGGGTGATCGCACAAC-3′, R: 5′-ATGCCTCGCTTAGTTGGCG-3′; Neuron Specific Enolase (*ENO2*, NM_001975.2), F: 5′-TATCCTGTGGTCTCCATTGAGG-3′, R: 5′-TTGCACGCTTGGATGGCTTC-3′; Kallikrein 3 (*KLK3*, NM_001648.2), F: 5′-ATTGAACCAGAGGAGTTCTTGAC-3′, R: 5′-AGCACACAGCATGAACTTGGTC-3′; Matrix metalloproteinase-9 (*MMP9*, NM_004994.2), F: 5′-GCACGACGTCTTCCAGTACC-3′, R: 5′-CAGGATGTCATAGGTCACGTAGC-3′; Prostate Tumour Overexpressed 1 gene (*PTOV1*, NM_017432.4), F: 5′-AACCTGGAGACCGACCAGTG-3′, R: 5′-TCTCTGTTGGTGAAGTGGAACTG-3′; *REST* (NM_005612.4), F: 5′-ATATGCGTACTCATTCAGGTGAG-3′, R: 5′-AATTGAACTGCCGTGGGTTCAC-3′; TUBB3 (NM_006086.4), F: 5′-CCTCTTCTCACAAGTACGTGCC-3′, R: 5′-CACATCCAGGACCGAATCCAC-3′. ACTB forward and reverse oligonucleotides (HK-SY-hu-600) were obtained from (PrimerDesign, UK). Oligonucleotides were validated prior to use by confirming they generated a single amplicon^51^.

### Quantitative PCR analysis (qPCR)

Reactions (20 µL) contained 25 ng cDNA, 300 nM forward and reverse oligonucleotide and 1x PrecisionPLUS qRT-PCR master mix (PrimerDesign), in RNAse/DNAse free DEPC-H_2_O. Triplicate reactions were performed using the StepOnePlus qRT-PCR system (Applied Biosystems, UK) under standard conditions. Control reactions contained cDNA from a reaction lacking reverse transcriptase (-RT) or cDNA replaced with nuclease-free water (no template control, NTC). Amplification of specific amplicons was determined via melt curve analysis^51^. Stable reference gene expression was determined via GeNorm analysis (PrimerDesign) from a panel of seven candidate reference genes and calculated using qBase + software (Biogazelle). *ACTB* was most stable under the experimental conditions and fold change in target gene expression was calculated via the 2^(−ΔΔCt)^ method^52^.

### Immunoblotting

Cell pellets were lysed in ice cold lysis buffer (50 mM Tris pH 8, 150 mM NaCl, 1 mM EDTA, 1% (w/v) Triton-X and 1% (v/v) Halt protease inhibitor cocktail (Thermo Fisher Scientific, UK)) on ice for 30 minutes and clarified by centrifugation at 16 000 × g for 5 minutes at 4 °C in a benchtop centrifuge. Protein concentrations were determined by Bradford assay^53^ and samples (1 µg/µl) were prepared in reducing sample buffer (final concentration: 5% (w/v) glycerol, 50 mM Tris pH 6.8, 1% (w/v) SDS, 2 mM EDTA, 0.2% bromophenol blue, 5% (v/v) β-mercaptoethanol) and denatured before use. Protein (10–30 µg) was resolved on SDS-PAGE gel then transferred to 0.45 µm nitrocellulose membrane (Millipore) following the method by Towbin^54^. Membranes were blocked with 5% (w/v) non-fat dried milk powder (Marvel) in PBS-Tween (0.1% v/v). Protein expression was analysed by immunoblotting with anti-AR (Santa Cruz Biotechnology, SC-816), anti-β-actin (Santa Cruz Biotechnology, sc-1615), anti-CD-44 (Cell Signalling Technology, #5640); anti-hASH1 (Abcam, Ab74065); anti-NSE (Abcam, Ab16808) or anti-PSA (1:200; Santa Cruz Biotechnology, sc-7638) at a dilution of 1:1000 dilution in 5% Marvel/PBS-Tween. Anti-mouse 800CW (926–32210), anti-goat 680LT (926–68024), anti-rabbit 800CW (926–32211) or anti-rabbit 680LT (926–68021) IRDye-conjugated fluorescent secondary antibodies were used at 1:10000 dilution in PBS-Tween containing 0.01% (w/v) SDS and analysed by Odyssey imaging system and ImageStudio 2.0 Software (LI-COR Biosciences, USA).

### Confocal microscopy

Cells were cultured under the treatment conditions outlined above. For longer incubations, prior to analysis, LNCaP cells were trypsinised and seeded at 2 × 10^4^ in 12 well plates containing glass cover slips and grown for the 2–3 days in media appropriate to the experiment. For shorter incubations, cells were also seeded directly into 6-well plates at 1 × 10^4^, and grown in media appropriate to the experiment. LNCaP cells were fixed with 4% paraformaldehyde in PBS, pH 7.4, for 20 minutes at room temperature. Coverslips were washed with PBS before cells were blocked and permeabilised in 20% v/v Goat Serum in PBS containing 0.2% (v/v) Triton-X for 1 hour at room temperature. Cells were incubated with hASH or NSE primary antibody (1:1000 in PBS containing 2% (v/v) goat serum and 0.2% (v/v) Triton-X) overnight at 4 °C then the appropriate fluorescent-conjugated secondary antibody (AlexaFluor488 (Thermo Fisher, A11029) or AlexaFluor568 (Thermo Fisher, A11008) at 1:1000 in PBS containing 2% (v/v) goat serum and 0.2% (v/v) Triton-X)) at room temperature, protected from light. Cells were mounted using VECTASHIELD containing DAPI (Vector Laboratories, UK). Representative images were captured using an LSM 880 Confocal microscope (Carl Zeiss Microscopy, UK) at 63x magnification. Primary and secondary antibody negative controls were also performed to confirm lack of non-specific staining (data not shown).

### Neurite outgrowth analysis

The length of neurite-like projections were quantified using Neurite Outgrowth Staining Kit (ThermoFisher Scientific, UK) according to manufacturer’s instructions and imaged via Confocal microscopy (LSM 880, Carl Zeiss Microscopy, UK). Images were captured from three separate regions and three representative slides using a 20x/0.8 objective lens and a 5 × 5 tile scan. The average cell body and neurite-like projection length were calculated by measuring 100 cell bodies or projections per tile scan image using Zen Blue 2.3 software (Carl Zeiss Microscopy, UK).

### Statistical analysis

Graphpad Prism 7.0 (Graphpad Software Inc.) was used to perform statistical analysis for qRT-PCR and neurite outgrowth experiments. Results are shown as the mean of three independent experiments ± SEM. Significance was determined using one-way or two-way ANOVA with the appropriate post-hoc testing (see figure legends) to identify specific treatment associated differences compared to control (untreated) cultures (p < 0.05).

## Supplementary information

Supplementary dataset 1

## Data Availability

All data generated or analysed during this study are included in this published article.
