# Supplementary material for: hASH1 nuclear localization persists in neuroendocrine transdifferentiated prostate cancer cells, even upon reintroduction of androgen
Source: Sci Rep. 2019 Dec 13;9:19076. doi: 10.1038/s41598-019-55665-y (PMC6911083; doi:10.1038/s41598-019-55665-y)

hASH1 nuclear localization persists in neuroendocrine transdifferentiated prostate cancer cells, even upon reintroduction of androgen

Jennifer A. Fraser[*], Joseph E. Sutton, Saba Tazayoni, Isla Bruce, Amy V. Poole

School of Applied Sciences. Edinburgh Napier University, Sighthill Campus, Edinburgh, EH11 4BN, UK.

[*]corresponding author; e.mail: j.fraser@napier.ac.uk

Supplementary information

Immunoblot images for Figure 1B

Androgen receptor immunoblot (upper band),

reprobed with actin (lower bands):

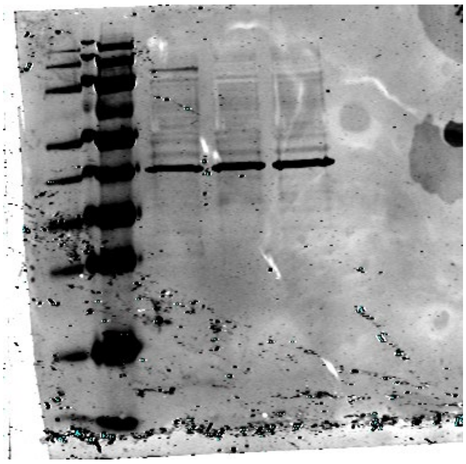

PSA immunoblot:

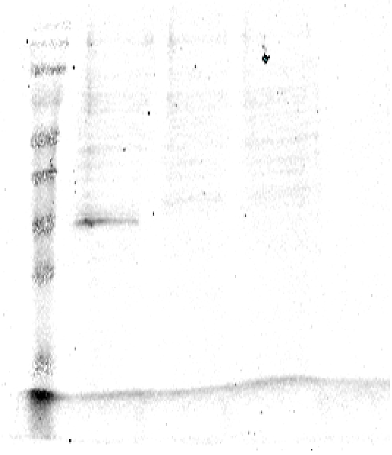

Immunoblot images for Figure 2B:

Androgen receptor:

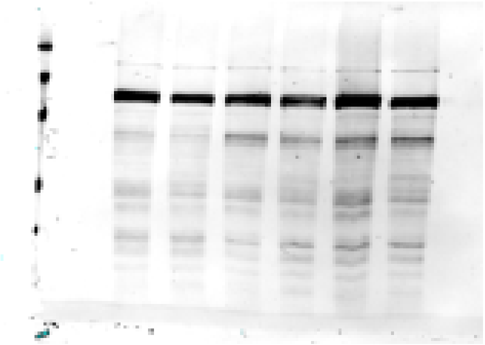

PSA:

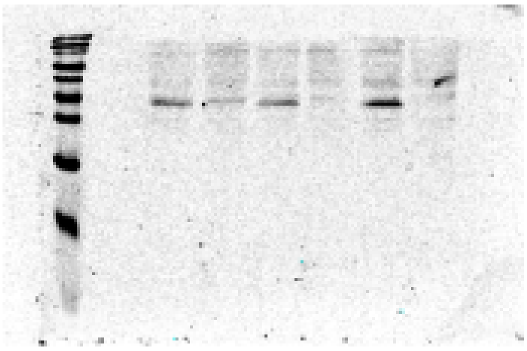

NSE:

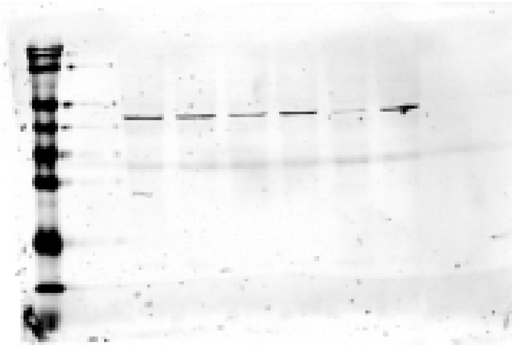

hASH1 immunoblot (lower bands), reprobed with Actin (upper bands):

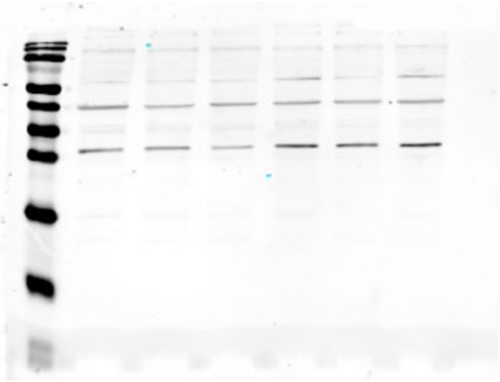

Immunoblot images for Figure 2D:

Androgen receptor:

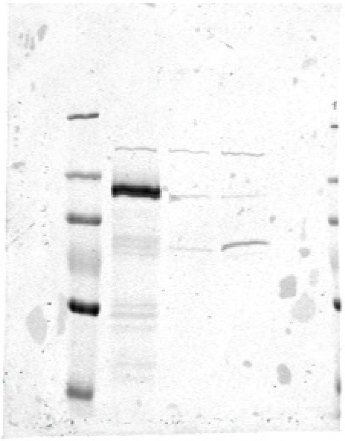

hASH1:

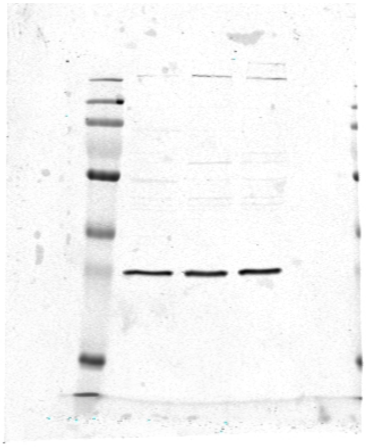

NSE:

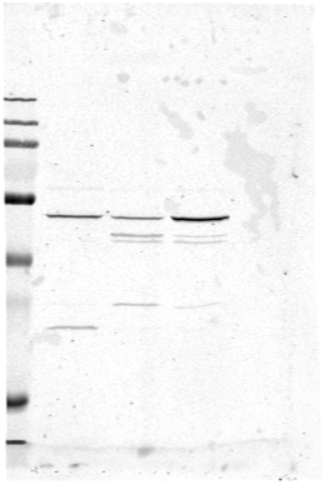

b-actin:

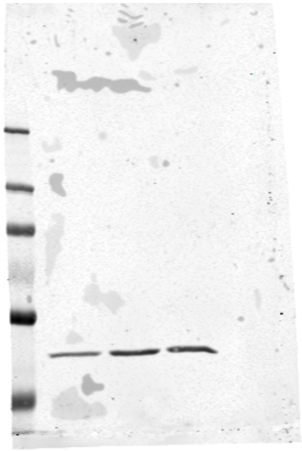

Immunoblot images for Figure 4D:

Androgen receptor:

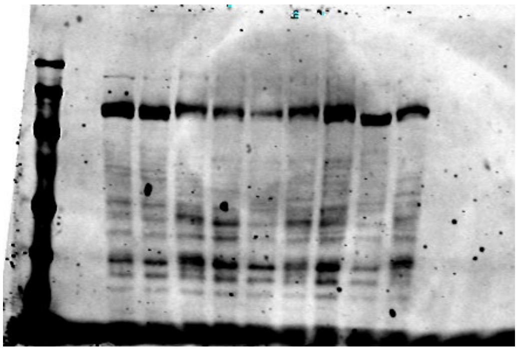

hASH1:

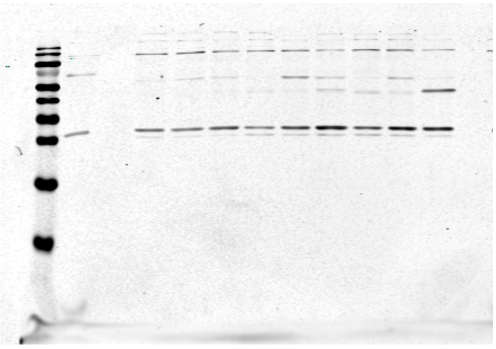

NSE:

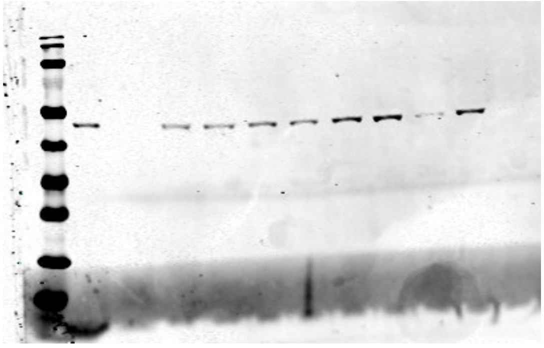

Actin:

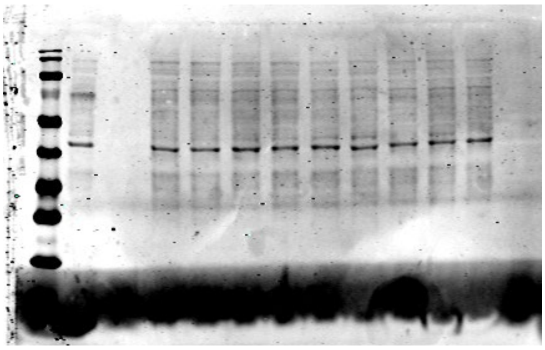

Immunoblot images for Figure 4E:

PSA:

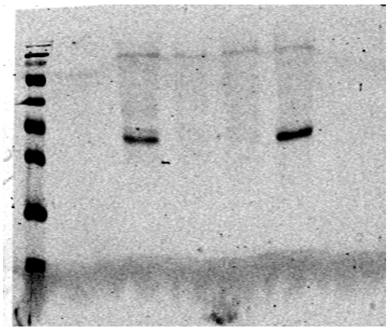

Actin:

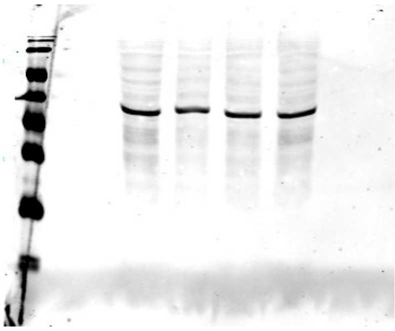

Immunoblot images for Figure 5C:

Androgen receptor:

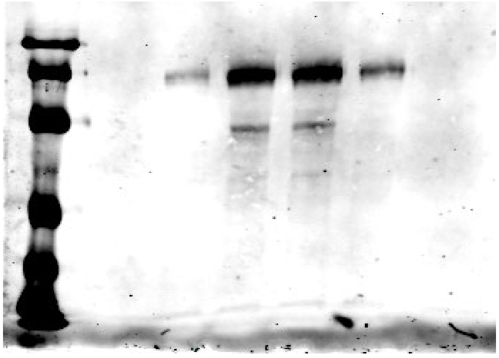

hASH1:

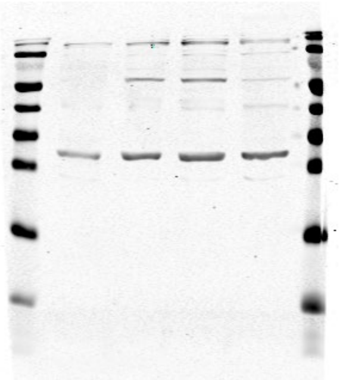

PSA:

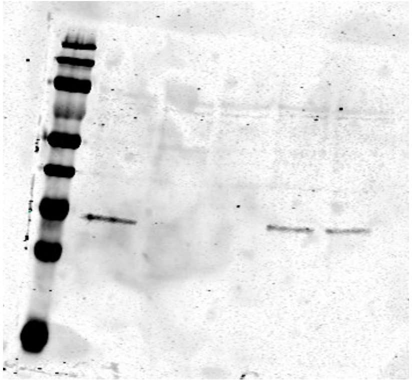

Actin (lower bands), reprobe of the androgen receptor blot:

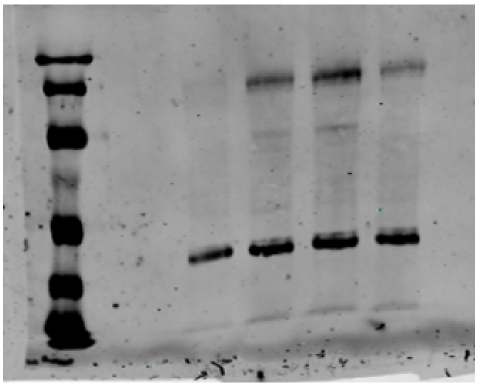

NSE:

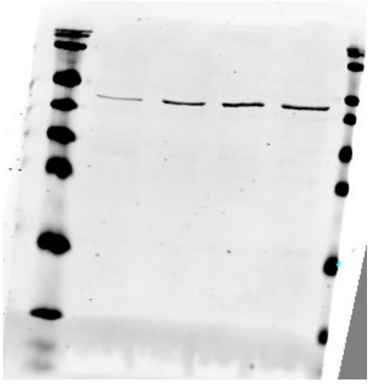

Images for Figure 6D:

Androgen receptor:

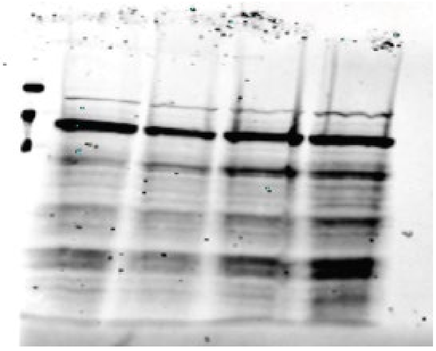

NSE:

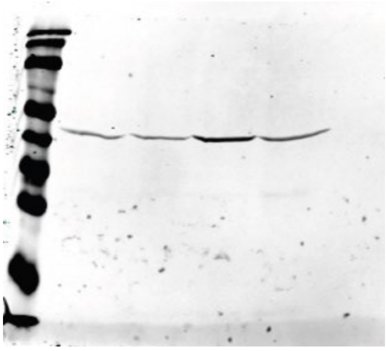

REST:

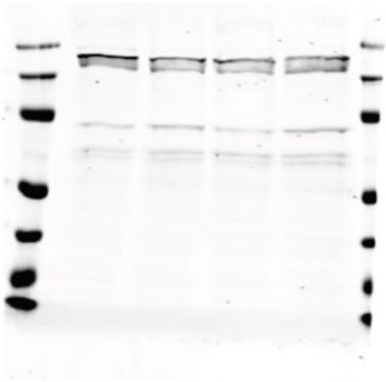

hASH1:

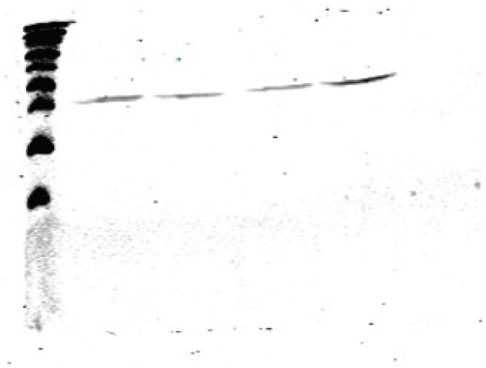

PSA:

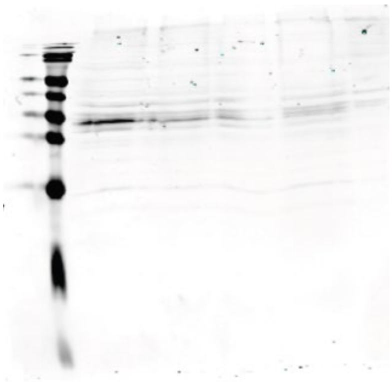

Actin:

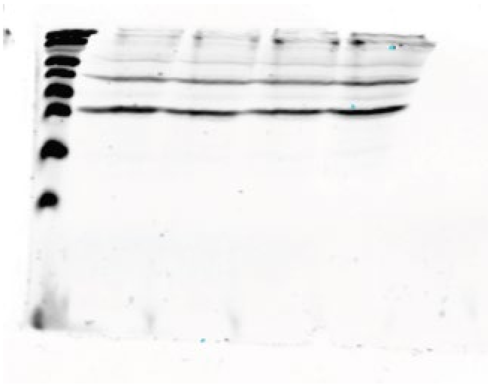

Supplement: Supplementary file 1 — Supplementary dataset 1 [file 41598_2019_55665_MOESM1_ESM.pdf]
